# Mercury hygiene and biomedical waste management practices among dental health-care personnel in public hospitals in Lagos State, Nigeria

**DOI:** 10.4314/ahs.v21i1.56

**Published:** 2021-03

**Authors:** John Oluwatosin Makanjuola, Uyi Idah Ekowmenhenhen, Lillian Lami Enone, Donna Chioma Umesi, Oladunni Mojirayo Ogundana, Godwin Toyin Arotiba

**Affiliations:** 1 Department of Restorative Dentistry, Faculty of Dental Sciences, College of Medicine, University of Lagos, Idi-Araba, Surulere, Lagos State, Nigeria; 2 Department of Preventive Dentistry, Lagos University Teaching Hospital, Idi-Araba, Surulere, Lagos State, Nigeria; 3 Department of Restorative Dentistry, Lagos State University Teaching Hospital, Ikeja, Lagos State, Nigeria; 4 Department of Restorative Dentistry, Lagos University Teaching Hospital, Idi-Araba, Surulere, Lagos State, Nigeria; 5 Department of Oral Biology and Maxillofacial Pathology, Faculty of Dental Sciences, College of Medicine, University of Lagos, Idi-Araba, Surulere, Lagos State, Nigeria; 6 Department of Oral and Maxillofacial Surgery, Faculty of Dental Sciences, College of Medicine, University of Lagos, Idi-Araba, Surulere, Lagos State, Nigeria; 7 Department of Oral and Maxillofacial Surgery, Lagos University Teaching Hospital, Idi-Araba, Surulere, Lagos State, Nigeria

**Keywords:** Biomedical waste management, mercury hygiene, dental personnel, Nigeria

## Abstract

**Background:**

Indiscriminate disposal of hospital wastes including mercury/amalgam wastes pose a serious threat to life and environment. There is a growing concern about biomedical waste (BMW) management among health care workers, however there are limited reports on BMW management by dental personnel in developing countries.

**Objectives:**

This study investigated the level of knowledge of BMW, observance of proper mercury hygiene and BMW management practice among public dental personnel in Lagos State, Nigeria.

**Methods:**

A cross-sectional study regarding BMW management across public hospitals in Lagos State, Nigeria was conducted following institutional ethics committee approval. A self-administered questionnaire was utilized to obtain data from different facilities selected by purposive and simple random sampling techniques as applicable. The questionnaires were distributed among 437 respondents by convenience sampling. The resulting data were statistically tested using Chi-square and G-test with p-value < 0.05 indicating significant level.

**Results:**

Amongst 437 respondents, majority were females (62.5%) and the highest proportion fell within the age range of 25–34 years (44.4%). Only 17.2% of the respondents had good knowledge of BMW management/legislation and 4.1% had good BMW practice. Less than half (49.4%) of respondents disposed mercury-contaminated materials inside the trash and majority (92.2%) did not observe proper mercury hygiene. Significantly better mercury hygiene practices were observed in secondary facilities (p=0.040).

**Conclusion:**

A minor proportion of public dental personnel had good knowledge and practice of proper mercury hygiene and BMW management. This shows there is an urgent need for training of health personnel on proper BMW handling and disposal in developing countries like Nigeria.

## Introduction

The innovation and advances in health institutions and science-based research has led to a rapid rise in generation of bio-hazardous waste at a disturbing rate. The accumulated increase in waste generation has caused serious threat to life and environment.^1^,^2^ It is therefore ironic that health systems which provide health care for the populace, also threaten the welfare of the same persons. Different authorities fail to enforce the relevant biomedical waste (BMW) management systems for a number of reasons such as inadequate professional training on waste disposal, limited financial resources and lack of appropriate waste management technologies.^3^

Hospital waste refers to biologic or non-biologic residual matter that is disposed and not to be re-used for any purpose. It is produced following diagnosis, patient treatments, biomedical research and laboratory procedures. 4,5 Dental waste is a subset of hospital waste and it is of two types- liquid waste and solid waste; each can further be categorized into risk (infectious waste and hazardous) and non-risk wastes.^6^–^8^ These infectious wastes contain different types of pathogenic micro-organisms while the hazardous wastes contain toxic metals.^8^,^9^ It has been reported that waste water from dental clinics typically contains raised levels of heavy metals such as mercury, silver, copper and zinc which arise mainly from placement/ removal of amalgam restorations and discarding of used radiographic fixer solution.^9^ Amalgam is the main hazardous solid waste used in dental clinics; its mercury content is recognized as a toxic element and it is the most volatile heavy metal known in nature.^6^,^10^,^11^ The management and handling rules of these BMW have been revised a number of times. The rule requires obligatory practice by health facilities to segregate the waste right from the source and then adopt the best disposal option that would protect the environment.^9^,^11^,^12^

There is a growing concern among dental personnel about the reported environmental effect caused by dental amalgam and necessary precautions are being taken to avoid mercury toxicity as well as reduce the release of environmentally harmful wastes from dental clinics.^13^,^14^ Following the Minamata Convention that is aimed at protecting human health as well as curbing the health hazards and environmental effect of mercury pollution, there is an agreement to phase-down globally and not to abruptly ban the use of dental amalgam until 2030 compared to the total ban of other mercury-containing products.^15^,^16^ The gradual phase-down is to encourage a smooth transition toward amalgam free dental practice.^17^

Despite the increasing global concern of use of dental amalgam, there are still reports in literature of improper mercury hygiene practices in developing countries.^18^–^20^ Currently, literature has reported that dental clinics contribute between 3% and 70% of environmental mercury.^21^–^24^ Poor mercury hygiene practices within the dental clinics are largely as a result of mercury spillage, improper storage of amalgam scraps and non-adherence to the necessary precautions during the placement and removal of amalgam restorations.^25^,^26^,^27^

Dental amalgam has a significant effect on the environment because even though each dental clinic contributes minimal amounts of mercury wastes to the environment, the cumulative amount generated by the entire dental profession has a hazardous impact on the environment.^28^,^29^ It is worth noting that metallic mercury is relatively harmless, however, when released to the environment, some bacteria species convert the mercury to organic methyl mercury which is a known neurotoxin. 30,31 This organic mercury enters the food web and steadily accumulates in higher organisms, particularly sea foods, and birds.^32^ Other portals of mercury release into the environment are through autoclaving of amalgam-filling dental instruments, incineration of amalgam wastes, uncontrolled disposal of extracted amalgam-restored teeth and amalgam waste in the regular municipal waste.^10^,^32^ The indiscriminate disposal of amalgam particles down the drain by dental personnel contributes to the mercury contamination in amalgam sludge/ waste water. This steady bio-accumulation of mercury released into the environment ultimately causes deleterious effects on the ecosystem.^10^,^12^,^28^,^32^

There are limited reports regarding the management of dental hazardous waste and the level of precautionary measures taken by dental personnel in African countries. Therefore, the purpose of this study was to assess the level of knowledge of BMW handling, observance of proper mercury hygiene and level of compliance with good BMW management practice among dental health care personnel in public hospitals in Lagos state, Nigeria.

## Methods

### Study description and Ethics

This descriptive cross-sectional study was based on collected data from dental health personnel working in primary, secondary and tertiary public dental health facilities in Lagos State, Nigeria. The proposal for this study was reviewed by the Lagos University Teaching Hospital Health Research and Ethics Committee and the study commenced after obtaining ethical approval (ADM/DCST/HREC/APP/2827). The research was conducted in full accordance with ethical principles including the World Medical Association Declaration of Helsinki (version 2008).

### Study setting and population

A list of 16 public dental facilities (primary, secondary and tertiary) in the 14 local government areas (LGA) that have dental facilities was obtained from the Nigeria Dental Association. One LGA had a primary facility, 2 had one tertiary facility each, while of the remaining 11 that had secondary facilities; two had the complement of having two secondary facilities each. A purposive sampling method was utilized to select the single facilities from the LGAs that had one facility each, while a simple random sampling technique by balloting was employed to select one facility each from the two LGAs that had two secondary facilities.

Consenting dental personnel who met the inclusion criteria in selected public dental facilities in Lagos State were recruited into the study. The inclusion criteria for the selection of participants included allied dental workers (consisting of dental nurses and dental therapists), 5^th^ (penultimate) or 6^th^ (final) year dental students, house officers, dental officers, specialists-in-training (junior and senior registrars) or consultants/ specialists who willingly gave their consent. Pre-clinical students and dental personnel not directly in contact with patients e.g. dental technologists were excluded from this study.

### Sample size calculation

The minimum sample size required for this study was calculated based on a previous study by Sanjeev et al^9^ who reported that 68.6% of dental health care personnel in Kothamangalam, India segregated BMW during disposal. The sample size was calculated by using the formula^33^: n = z2pq / d2, where n = the minimum sample size; z = standard normal deviate corresponding to the level of significance at 95% confidence interval = 1.96; p (the proportion of the target population estimated to have a particular characteristic from previous study) = 0.686; q = 1.0 – p = 1-0.686= 0.314 and d = degree of accuracy desired, set at 0.05. Therefore, n was = (1.96x1.96x0.686x0.314 / 0.05x0.05) = 331. To compensate for attrition and to increase the power of study, the required sample size for this study was increased to 437.

### Selection of participants

Following the selection of health facilities by non-probability and probability sampling techniques, the participants were selected in each facility by convenience sampling method. Convenience sampling was used to select members of the target population that were easily accessible and available at a given point in time and willing to participate in the study.

### Questionnaire design

A well-structured questionnaire was designed by the researchers. The compact nature of the formatting and the use of closed-questioning in the majority was introduced to increase compliance. The questionnaire was primarily designed to obtain information about knowledge of BMW generation and information about the waste management i.e. procedures used for disposal of waste in and from dental clinics by the health care personnel in public hospitals. The questionnaire began with an introductory explanation of the purpose of the study, and anonymity of each respondent was emphasized. The first section of the questionnaire consisted of questions on demographics and professional characteristics related to respondent's age, sex, qualification, and clinic location. The other sections of the questionnaire were designed to collect information on: (a) level of knowledge and attitude on amalgam waste and other BMW generation and legislation, (b) dental clinic environment, dental practice and mercury hygiene practices, (c) BMW management practice. A pretesting of the designed questionnaire was conducted among fifteen dental personnel comprising of five dental students, five dentists across the different cadres and five dental nurses. These 15 respondents completed the initial questionnaire designed for the study and were able to help indicate areas of unclear or ambiguous questions. They found the questionnaire easy to read, appropriate, and not excessively demanding. The primary outcome measures of this study were level of knowledge of BMW generation and legislation (comprising of 10 questions), rating of respondents mercury hygiene practices (comprising of 11 questions) and rating of respondents' BMW management practices (comprising of 10 questions). The participants' responses in the different sections were graded as poor or good. Grades were assigned based on the percentage of overall correct answers; based on < 70% and ≥70% defined as poor and good grades, respectively.

### Data collection

The purpose of the study was explained to each of the respondents that met the inclusion criteria in the selected hospitals and thereafter, a signed consent form was obtained before being included in the study. Each participant was given a copy of the questionnaire personally by one of the investigators. The questions were explained to avoid any ambiguity and they were thereafter requested to answer it as promptly as possible. Four hundred and thirty seven questionnaires were retrieved following distribution of questionnaires. Confidentiality was maintained by giving codes for reference to the participants.

### Statistical analysis

The collected data was checked for consistency and completeness. It was then coded in a database for analysis. Data collected was analyzed using SPSS software version 22.0 (IBM Corp, Armonk, NY, USA). Frequency distribution tables and cross-tabulations were generated from the responses provided. Shapiro-Wilk's normal distribution test was used to determine the normality of quantitative variables while statistical associations between categorical variables were determined using Pearson's Chi-square tests and G likelihood- ratio test. For all statistical tests, probability values less than 5% inferred the criterion for statistical significance.

## Results

Four hundred and thirty seven dental health care personnel were included in our study. Most respondents, 273 (62.5%), were females and a greater proportion of all respondents, 194 (44.4%), fell within the 25–34 years age group with a mean age (±sd) of 28.92 (±8.02) years. One hundred and thirty six (31.1%) were dental students whilst 111 (25.4%) were allied dental workers and 190 (43.5%) were dental practitioners across different cadres. The median (inter-quartile range) of the respondents' work experience was 4.0 (2.0 – 10.0) years (Table 1).

**Table 1:** Socio-demographic characteristics of participants

Variable	Frequency (n=437)	Percent (%)
**Age group**		
<25 years	151	34.6
25 – 34 years	194	44.4
35 – 44 years	63	14.4
45 – 54 years	22	5.0
55 – 64 years	7	1.6
Mean(±sd) = 28.92(±8.02)		

**Gender**		
Male	164	37.5
Female	273	62.5

**Cadres**		
Students	136	31.1
Allied Workers	111	25.4
Dental Practitioners	190	43.5

**Cadres Profile**		
Dental Nurses	74	16.9
Dental Therapists	37	8.5
500 Level Students	57	13.0
600 Level Students	79	18.1
House Officers	74	16.9
Dental Officers	34	7.8
Junior Registrars	37	8.5
Senior Registrars	25	5.7
Consultants/ Specialists	20	4.6

**Place of work**		
Primary Health Care Centre	2	0.5
Secondary Facility	62	14.2
Tertiary Institution	237	54.2
Dental School	136	31.1

**Years of experience**		
0 (Students)	136	31.1
< 5	161	36.8
5 – 10	74	16.9
11 – 20	53	12.1
21 – 35	13	3.0
Median(inter-quartile range) = 4.0 (2.0 – 10.0)		

n= total number of respondents

sd= standard deviation

About one-third of respondents (164, 37.5%) knew that amalgam belonged to category of hazardous wastes and 344 (78.7%) felt there was a significant need to label (colour code) waste containers in the clinics. Two hundred and sixteen (49.4%) knew the universal symbol of biohazard waste while the remaining 221 (50.6%) identified biohazard waste wrongly as either harmful, flammable, harmful to the environment or fatal. Majority of respondents, 238 (54.5%), did not know whether amalgam scraps and lead foils of X-ray films were handed over to waste management for recycling purposes. Three hundred and thirty five (76.7%) respondents were unaware of existing Nigerian environmental regulations, legislation, medical waste management policy or guidelines of mercury disposal and only 40 (9.2%) knew that BMW should not be stored for more than 48 hours. A greater proportion of respondents, 343 (78.5%), had no previous training in BMW management and 370 (84.7%) felt BMW management is an urgent issue that must be addressed in Nigeria. Overall, majority of respondents, 362 (82.8%), had poor knowledge on BMW management/ legislation (Table 2).

**Table 2:** Knowledge level of mercury/biomedical waste (BMW) generation and legislation

Knowledge about Biomedical Waste Handling	Frequency	(%)
**Categorization of amalgam waste**		
Hazardous*	164	37.5
Chemical	149	34.1
Black bag	19	4.3
Non-risk	12	2.7
Infectious	9	2.1
Trash	16	3.7
Don't know	68	15.6
**Knowledge on colour-coding segregation of BMW**		
Yes	225	51.5
No	212	48.5
**Perception on lack of awareness regarding existing health care waste** **management services in Nigeria**		
Yes	328	75.1
No	109	24.9
**Awareness of existing Nigerian environmental regulations, legislation, BMW** **management policy or guidelines of mercury disposal**		
Yes	102	23.3
No	335	76.7
**Maximum hours for storage of waste according to the BMW (Management** **and Handling) rules**		
48 hours*	40	9.2
12 hours	102	23.3
72 hours	13	3.0
96 hours	2	0.5
Don't know	280	64.1
**Safe management of health care waste is an urgent issue that must be** **addressed in Nigeria**		
Agree*	370	84.7
Disagree	4	0.9
Don't know	63	14.4
**Waste management is team work**		
Agree*	383	87.6
Disagree	9	2.1
Don't know	45	10.3
**Previous training in BMW management**		
Attended	94	21.5
Not attended	343	78.5
**Objective assessment of knowledge levels on BMW generation, handling and** **legislation based on answers provided**		
Good	75	17.2
Poor	362	82.8

* Correct answer

About one-third of respondents, 148 (33.9%), reported that the air conditioner filters were periodically cleaned in the clinics where they worked and only 42 (9.6%) reported that the mercury vapour levels were measured periodically. Four hundred and two (92%) reported the absence of mercury spill kit in the dental clinic. Dental amalgam was still used as a restorative material by 372 (85.1%) of the respondents and 51 (11.7%) respondents were unaware of the precautions to be taken during the placement and removal of amalgam restorations. Most respondents, 244 (55.8%) were not aware of the site of drainage of waste contents in their clinics and 189 (43.2%) disposed of amalgam scrap in the trash. No respondent had used amalgam separator before. Majority of our respondents, 403 (92.2%), did not observe good mercury hygiene in their practice (Table 3).

**Table 3:** Dental Clinic environment, dental practice and mercury hygiene practices

Mercury Handling and Disposal Practice	Frequency	(%)
**Periodic cleaning of A/C filter**		
Yes*	148	33.9
No	289	66.1

**Periodic clinic monitoring of mercury vapour level**		
Yes*	42	9.6
No	395	90.4

**Mercury spill kit availability in the clinic**		
Present*	35	8.0
Absent	402	92.0

**Use of amalgam for restoring carious/defective tooth**		
No*	65	14.9
Yes	372	85.1

**Removal of old/defective amalgam restoration**		
Yes	356	81.5
No	81	18.5

**Precautions taken during placement and removal of amalgam restorations (Multiple** **answers)**		
Wearing face mask, eye goggles, hair caps and clinical coats*	313	71.6
Ensuring adequate amalgamation*	269	61.6
Ensuring proper draping of patient*	206	47.1
Attempt to section and scoop out amalgam restoration on removal*	113	25.9
Removal of old amalgam fillings using water spray*	86	19.7
Use of rubber dam isolation technique and inspection of mucosa on removal*	71	16.2
Unaware of the necessary precautions stated above	51	11.7

**Evacuation method used in practice**		
High-volume evacuation*	44	10.1
Saliva ejector	216	49.4

**Place of storage for leftover amalgam scrap**		
Disposed in the hazardous waste bag*	100	22.9
Regular dustbin/trash	189	43.2
Empty bottle	12	2.7
Bottle with water	13	3.0
Bottle with radiographic fixer	4	0.9

**Extraction of amalgam restored teeth**		
Yes	392	89.7
No	45	10.3

**Site of disposal of extracted amalgam restored teeth**		
Segregated as hazardous waste*	222	50.8
Regular dustbin	168	38.4
Recycle	2	0.5

**Objective assessment of mercury hygiene practice based on answers provided**		
Good	34	7.8
Poor	403	92.2

* Correct practice

Two hundred and twenty eight (52.2%) respondents segregate the BMW in the clinic and 376 (86%) dispose of sharp disposables inside sharp boxes. A greater proportion of respondents, 216 (49.4%), dispose amalgam contaminated gloves/ gauze into the regular dustbin and 185 (42.3%) dispose hazardous liquid waste into the regular drain. Only 7.3% of our respondents strictly adhered to the manufacturer's recommendations when discarding the developer and fixer solutions in the clinic. Out of the 437 respondents, 18 (4.1%) had good BMW management practice (Table 4).

**Table 4:** Biomedical waste management practices

Biomedical Waste Handling and Disposal Practice	Frequency	(%)
**Frequency of cleaning dental suction unit in the clinic**		
Daily*	184	42.1
Twice weekly	7	1.6
Once weekly	27	6.2
Once monthly	22	5.0
Once yearly	8	1.8
Don't know	189	43.2

**Practice of using colour coded containers to dispose biomedical waste**		
Segregation of waste*	228	52.2
Not done	209	47.8

**Disposal of sharp disposables in the clinic**		
Inside the sharps box*	376	86.0
Garbage/Regular dustbin	48	11.0
Along with other biomedical wastes	13	3.0

**Handing over of amalgam scraps and lead foils of X-ray films to waste** **management for recycling purposes**		
Done*	63	14.4
Not done	374	85.6

**Digital radiography use in the clinic**		
Always*	99	22.7
Often	62	14.2
Sometimes	113	25.9
Rarely	66	15.1
Never	97	22.2

**Discard process for developer or fixer solution in the clinic**		
Strict adherence to manufacturer's recommendations*	32	7.3
Mix and discard into drain	117	26.8
Send for recycling	15	3.4
Don't know	250	57.2

**Site of disposal for excess mercury and amalgam contaminated gauze and** **gloves**		
Segregated as hazardous waste*	99	22.7
Garbage/Regular dustbin	216	49.4
Drain	47	10.8
Plastic bags	38	8.7
Store in glycerin	3	7.0
Unaware of site of disposal	34	7.7

**Disposal practice for hazardous liquid waste**		
Chemical treatment and discharge into drains*	85	19.5
Into the drain	185	42.3
Don't know	139	31.8

**Contacting a certified waste carrier service for recycling or disposal of** **hospital waste**		
Yes*	136	31.1
No	301	68.9

**Objective assessment of biomedical waste management practice based on** **answers provided**		
Good	18	4.1
Poor	419	95.9

* Correct practice

Majority of respondents in the years-of-experience category (from nil years to 21–35 years of experience) had poor knowledge of BMW generation/legislation. There was a significant difference (p=0.01) when comparing knowledge in years-of-experience category. Majority of respondents had poor mercury hygiene practice in the primary, secondary, tertiary health facilities and dental school (100%, 83.9%, 92.4% and 95.6% respectively); although significantly better practices were observed in general (secondary) hospitals (p=0.012). Results also revealed poor BMW management practice which was statistically significant when comparing the practice of different cadres (p=0.006), place of work (p=0.006) and practice across the different years-of-experience (p=0.001) (Table 5). The majority of respondents who had good knowledge of mercury hygiene and good knowledge of BMW management had poor practice of the same (82.7% and 92% respectively). A look at good mercury hygiene practice showed a statistically significant difference between the 17.3% respondents who had good knowledge of mercury hygiene compared to 5.8% respondents who had poor knowledge (p=0.001) (Table 6).

**Table 5:** Knowledge of biomedical waste generation and legislation, mercury hygiene practices and practice of biomedical waste management among the different cadres of participants

	High knowledge of biomedical waste generation and legislation	Low knowledge of biomedical waste generation and legislation	Good mercury hygiene practice	Poor mercury hygiene practice	Good biomedical waste management practice	Poor biomedical waste management practice
**Age (years)**						
< 25	18(11.9)	133(88.1)	10(6.6)	141(93.4)	3(2.0)	148(98.0)
25 – 34	34(17.5)	160(82.5)	17(8.8)	177(91.2)	9(4.6)	185(95.4)
35 – 64	23(25.0)	69(75.0)	7(7.6)	85(92.4)	6(6.5)	86(93.5)
	χ^2^=6.911	p=0.032*	χ^2^=0.547	p=0.761	χ^2^=3.216	p=0.200
**Gender**						
Female	32(19.5)	132(80.5)	15(9.1)	149(90.9)	8(4.9)	156(95.1)
Male	43(15.8)	230(84.2)	19(7.0)	254(93.0)	10(3.7)	263(96.3)
	χ^2^=1.020	p=0.313	χ^2^=0.683	p=0.409	χ^2^=0.383	p=0.536
**Cadres**						
Students	15(11.0)	121(89.0)	6(4.4)	130(95.6)	1(0.7)	135(99.3)
Allied workers	15(13.5)	96(86.5)	12(10.8)	99(89.2)	12(10.8)	99(89.2)
Dental practitioners	45(23.7)	145(76.3)	16(8.4)	174(91.6)	7(3.9)	183(96.2)
	χ^2^=10.998	p=0.027*	χ^2^=4.439	p=0.350	χ^2^=14.584	p=0.006*
**Place of work**						
Primary and Secondary Facilities	15(23.4)	49(76.6)	10(15.6)	54(84.4)	5(7.8)	59(92.2)
Tertiary Institution	45(19.0)	192(81.0)	18(7.6)	219(92.4)	13(5.5)	224(94.5)
Dental School	15(11.0)	121(89.0)	6(4.4)	130(95.6)	0(0)	136(100)
	χ^2^=6.724	p=0.151	χ^2^=12.896	p=0.012*	G=14.556	p=0.006*
**Years of experience**						
0 (Students)	15(11.0)	121(89.0)	6(4.4)	130(95.6)	0(0)	136(100)
< 5	24(14.9)	137(85.1)	15(9.3)	146(90.7)	8(5.0)	153(95.0)
5 – 10	17(23.00)	57(77.0)	7(9.5)	67(90.5)	7(9.5)	67(90.5)
11 – 35	19(28.8)	47(71.2)	6(9.1)	60(90.9)	3(4.5)	63(95.5)
	χ^2^=13.271	p=0.01*	χ^2^=4.023	p=0.403	G=15.707	p=0.001*

χ^2^ = Pearson's Chi-square test

* Statistical significance; p<0.05

G = G-likelihood-ratio test

**Table 6:** Knowledge versus mercury hygiene practice and biomedical waste management practice among the different cadres of participants

	High knowledge	Low knowledge
**Mercury hygiene practice**		
Good	13 (17.3)	21 (5.8)
Poor	62 (82.7)	341 (94.2)
	χ^2^=11.516	p=0.001*

**BMW management practice**		
Good	6 (8.0)	12 (3.3)
Poor	69 (92.0)	350 (96.7)
	χ^2^=3.453	p=0.063

χ^2^ = Pearson's Chi-square test

* Statistical significance; p<0.05

## Discussion

The growing global concern has brought to focus the improper BMW disposal practices in developing countries worldwide. Despite the small amount of wastes generated by dental clinics compared to other medical facilities, the poor handling and disposal practices by the dental health personnel cannot be overlooked because it is still contributory to the health threat posed to man, wildlife and the environment.^18^,^20^ There has been a rise in the number of hospitals and private clinics resulting from the progressive growth of the population as well as urbanization. This has led to a rise in BMW generation which poses a threat to human health.^34^ Despite the growing concern among dental practitioners about BMW management, there are limited reports on dental hazardous waste management in African countries among dental health care personnel.^35^

Our study showed only 17.2% had good knowledge of BMW generation and legislation which was similar to previous studies which revealed that the majority of the Indian medical and dental professionals were not aware of the proper clinical waste generation, regulations, legislations and management.^36^,^37^ This shows that great effort is still required of health institutions and the government to create more awareness and properly orientate health care personnel on standard BMW management practices.

Our results show that the mercury hygiene and disposal practice of hazardous wastes by dental health care personnel are poor. Our study results show that dental staffs, municipal workers and the larger population are exposed to hazardous health risk and that their practices could also be detrimental to the balance of the ecosystem. Another Nigerian study reported similar findings of a poor compliance with some of the standard mercury hygiene practices such as the use of rubber dam, high volume suction, and water cooling when removing or polishing amalgam restorations among Nigerian dentists.^35^

Based on the findings of our study, the majority of respondents did not know if the air conditioner filters were periodically cleaned in their place of work and they reported that the mercury vapour level was not measured periodically in their clinic environment. This buttresses the findings of a previous study where an unacceptably high level of mercury vapour was measured in a Nigerian Restorative Dental Clinic.^38^ On the contrary, another study carried out among Indian dentists reported that majority of the dentists were concerned about the periodic changing of air conditioner filters, however, they were also particularly deficient in periodic monitoring of mercury vapour at the clinics.^39^ The FDI World Dental Federation recommends that the dental operatory be monitored periodically, preferably annually, or after a mercury spill clean-up.^40^ The dental clinic environment and mercury hygiene practices in the United States (US) is governed by the Occupational Safety and Health Administration (OSHA) and the current OSHA permissible exposure limit (PEL) for mercury vapour is 0.1 mg/m^3^ of air as a ceiling limit. One of their guidelines is that at no time should the mercury vapour exceed the ceiling level. However, this ceiling PEL is generally not reflective of the dental practitioners' real-time exposure. The preferred safety standard is the US Environmental Protection Agency's Reference concentration for Chronic inhalation exposure which is the same as the United Kingdom's Occupational Exposure Standard (at 25 µg/m^3^ air for 8 hours a day, 40 hours per week) and this is measured based on a personal dosimetry.^41^,^42^ Nigerian safety and environmental protection agencies have no existing legislation or regulation regarding the permissible mercury exposure limit of health workers, monitoring or focusing on air quality and the safe mode of managing mercury spills at the dental clinics.

Despite the global call to phase-down and to eventually phase-out amalgam in the near future, amalgam was still used by 85.1% of the respondents and 11.7% of our respondents were unaware of the precautions to be taken during the placement/ removal of amalgam restorations. A Nigerian study by Umesi et al^43^ among dental students and dentists showed that they were still placing a significantly large percentage (57.5%) of amalgam restorations to restore carious teeth. The phasedown campaign in Nigeria needs to be intensified to encourage the discontinuation of amalgam in the dental clinics.

The current findings of this study revealed that majority of respondents did not utilize amalgam separator or segregate amalgam wastes, instead, they disposed amalgam scraps in the general trash. This will result in the release of the mercury vapours into the air since garbage is usually burnt on regular daily basis in an open site. Furthermore, the accumulation of dental waste, amalgam scraps and amalgam contaminated products in the landfill over a period of time will lead to water and soil contamination.^10^,^28^,^29^ Unfortunately, the accumulated effects of such environmental burdens are often ignored.^10^ This shows there is an urgent need to address this issue because it poses considerable danger to human health and the environment. Extensive training of dental health personnel is also needed to promote good mercury hygiene practices among these health personnel especially during the replacement of defective amalgam restorations.

Majority of respondents (95.9%) had poor BMW management practice. Even though 42.1% of the respondents reported that the dental suction unit is cleaned daily and 52.2% use colour coded bags to dispose the waste, majority of the disposal practices were not routinely performed. Majority of respondents, 57.2% were not aware of how developer and fixer solutions were discarded and 26.8% confirmed the solutions were mixed and discarded into the drain. From our findings, majority of the respondents dispose excess mercury and amalgam contaminated gauze and gloves straight into the garbage/regular waste disposal bin. Our findings are similar to that of another study where 36% of their respondents clean the suction units daily and 67% segregated their waste, however, 45% of their respondents disposed spent amalgam capsules in the garbage and 54% also dispose amalgam scraps in the trash.^11^ Thirty-four percent of dentists in their survey were draining the fixer into the washbasin, 60% were of the opinion that developer can be flushed down the drain, and 25% were of the view that spent developer and fixer solutions be mixed and flushed into the drain. Another study that evaluated dental waste management reported that used radiographic processing solution was disposed off in the drain in all clinics and the lead foil that protects the X-ray film was discarded in the regular waste disposal bin.^6^ The majority of amalgam waste was also disposed in the garbage or drain. They pointed out that the major setback leading to the poor disposal practice of discarding radiograph processing solutions into the drain in Palestinian Dental Clinics are as a result of lack of recycling companies or silver recovery units.^6^ This is also the case in Nigeria and this is reinforced by our findings where 55.6% of our respondents reported that they were unaware of a certified waste carrier service for recycling.

The present study showed that there was poor mercury hygiene practice among dental personnel and secondary facilities appeared to demonstrate better practices than primary or tertiary institutions. There was also a significant difference when comparing level of knowledge and practice based on years-of-experience category; dental personnel with more years of experience had better knowledge and practice of BMW management. These significant differences between different groups with respect to the mercury hygiene practice, knowledge and practice of BMW management is expected because the years of experience and place of work most likely have a significant impact on the knowledge and practice of BMW management.^44^ Our result findings also revealed that majority of respondents that had high knowledge of mercury hygiene and BMW management still had poor practice. This shows that high knowledge does not necessarily translate to good practice.

The main basis for dental waste management in the European Union is the Waste Framework Directive that requires member states to execute actions of appropriate waste management without causing harm to human health or having any negative impact on the environment. 45 In Nigeria and several African countries on the other hand, there are no set guidelines for BMW management and these waste handling have not received adequate attention. These developing countries lack a comprehensive legislation and have unauthorized scrap yards. Unfortunately, BMW are still handled and disposed alongside domestic/municipal wastes and this endangers the health of municipal workers, the public and the environment at large.^45^–^47^ The existing challenges of BMW management in Nigeria are- inappropriate storage practices, routine dumping of infectious and hazardous waste with municipal waste, unsatisfactory labeling of hazardous waste and poor awareness about the management of medical waste.^48^ However, the findings of a survey also carried out in Lagos State, Nigeria revealed that six out of the seven hospitals managed their BMW by waste segregation, collection/on-site transportation, on-site storage and off-site transportation. The wastes of the surveyed hospitals were mainly treated using hydroclave and rarely by incineration. It was noted that Lagos State has been more effective than other parts of Nigeria as regards medical waste management by conducting intervention programs to ensure compliance and safety of waste management processes; and by the construction of several well-equipped transfer loading stations available at different sites within the state.^48^

Without the presence of set national legislation, regulations and services, amalgam and other hazardous wastes will keep being disposed in the trash and sewer systems. The formulation, implementation and adherence of environmental regulations as well as national waste disposal guidelines that addresses the various categories of dental waste is key to tackling this problem. This national collaborative effort will reduce the hazardous effects of such waste to the barest minimum or possibly eliminate it. The results of this present study provides the hospital authorities with data upon which they can develop a strategy for improving BMW management. Based on our findings, we propose that universities should compulsorily integrate BMW management and education of the hazards associated with improper waste disposal as part of undergraduate curriculum for dental students, dental nurses and therapists. Also, government hospitals should organize continuing medical education and extensive training programmes for all health care staff to update existing knowledge about mercury hygiene and BMW management. The hospital staff should also be educated that managing BMW is a team work. In addition, it is highly recommended that regular monitoring and quality control activities should be introduced and strengthened in hospitals to ensure effective and satisfactory BMW practices among hospital staff. Finally, central treatment plants and recycling companies should be introduced for all BMW at restricted sites of each state. This would be beneficial for health care facilities, thereby, limiting the number of collection sites.

## Conclusion

It can be concluded from our study that there is low level of knowledge about BMW generation hazards, legislation and management as well as laxity in performing standard mercury-hygiene and BMW management practices among dental health care personnel in Lagos State, Nigeria. It is hoped that these findings will spur further investigations by other researchers in Nigeria as well as in other developing countries regarding generation, handling and disposal of dental and medical waste. This will provide comprehensive data so that decisive actions can be implemented towards ensuring an efficient mercury hygiene and BMW management system throughout Nigeria and other developing countries. The establishment of a comprehensive protocol for BMW management is imperative in Nigeria.

## Data Availability

The data that support the findings of this study are available from the corresponding author upon reasonable request.
